# Umbilical Cord Hematoma: A Case Report and Review of the Literature

**DOI:** 10.1155/2018/2610980

**Published:** 2018-03-26

**Authors:** Gennaro Scutiero, Bernardi Giulia, Piergiorgio Iannone, Luigi Nappi, Danila Morano, Pantaleo Greco

**Affiliations:** ^1^Department of Morphology, Surgery and Experimental Medicine, Section of Obstetrics and Gynecology, Azienda Ospedaliero-Universitaria S. Anna, University of Ferrara, Via Aldo Moro 8, 44121 Cona, Ferrara, Italy; ^2^Department of Medical and Surgical Sciences, Institute of Obstetrics and Gynecology, University of Foggia, Viale L. Pinto, 71100 Foggia, Italy

## Abstract

**Objectives:**

To deepen the knowledge in obstetrics on a very rare pregnancy complication: umbilical cord hematoma.

**Methods:**

A review of the case reports described in the last ten years in the literature was conducted in order to evaluate epidemiology, predisposing factors, potential outcomes, prenatal diagnosis, and clinical management.

**Results:**

Spontaneous umbilical cord hematoma is a rare complication of pregnancy which represents a serious cause of fetal morbidity and mortality. There are many risk factors such as morphologic anomalies, infections, vessel wall abnormalities, iatrogenic causes, and traction or torsion of the cord, but the exact etiology is still unknown.

**Conclusions:**

Due to the rarity of this condition, every new case of umbilical cord hematoma should be reported in order to improve the knowledge of predisposing factors, prenatal diagnosis, and clinical management.

## 1. Introduction

Although very rare, umbilical cord hematoma (UCH) is a real serious complication of pregnancy. It represents a rare cause of acute fetal distress that may be shown by the decrease of fetal movement or fetal death [1].

Lately, a case of UCH resulted in perinatal death at our department stimulating our interest in performing this review of the literature, emphasizing the research on pathogenesis, diagnosis, and management for UCH.

The study by Dipple et al., with 36 cases, is the largest series published on this topic so far. It estimates an incidence rate of 1 in 5505. Although umbilical cord complications may be the second most common cause of stillbirth [2], umbilical cord hematoma has been reported as a rare cause for stillbirth and fetal distress; the overall perinatal loss rate was approximately 50%, and the incidence of this disorder in live births would then be approximately one in 11,000 pregnancies.

Our review of the English literature resulted in 9 publications of 11 cases of UCH in the years 2008–2017 [3–11]. Of the 11 cases of spontaneous UCH reported in the 9 studies published in the last 10 years, 2 were stillbirths, 1 occurring antenatally and 1 on day 6 of life [6, 8]. Of the nine live born cases, 7 presented at term of gestational age, 2 preterm [3–5, 7, 9–11].

## 2. Materials and Methods

A review of the literature was conducted in order to identify the case reports reported in the English language. We searched PubMed MEDLINE electronic database published between 2008 and 2017 on https://www.ncbi.nlm.nih.gov/pubmed. The keywords used were as follows: “Umbilical,” “Cord,” and “Haematoma.” Different combinations of the terms were used. Moreover, references in each article were searched to identify potentially missed studies. We chose 2008 as a starting year point for our literature search because this year was marked by a review of the literature by Gualandri et al. [12] for the years 1958 to 2008. From the authors' descriptions of individual case reports, we took available and reliable information about the possible predisposing factors, clinical presentation, diagnosis, and management (Table 1). Because of the lack of uniformity in the cases reviewed, we have not made any calculations with statistical significance.

## 3. Results

### 3.1. Pathogenesis

The exact etiology of UCH still remains unexplained. Many theories have been proposed but without final results. Probably a combination of different factors leads to UCH.

Risk factors for spontaneous umbilical cord hematoma are various. They include morphologic anomalies of the umbilical cord (both in length and in thickness), true knots, cord prolapse, traction or torsion, velamentous insertion of the cord, vessel wall abnormalities, umbilical cord cysts, abdominal trauma in pregnancy, postterm pregnancy, infections (chorioamnionitis and funisitis), deficiency of Wharton's jelly, congenital defects, and many more remain unexplained [13]. Fetal hypoxia and anemia may occur due to the compression of the umbilical vessels leading to perinatal asphyxia and stillbirth. Iatrogenic causes secondary to amniocentesis, in utero transfusions and diagnostic cordocentesis are also reported [14].

Spontaneous bleeding in the umbilical cord is due to a disruption of the vessel wall through which, in most cases, an extravasation of blood into Wharton's jelly occurs. [15]. A high intravascular pressure could be implicated in its formation. The hematoma can compromise the maternal-fetal circulation by compressing the vessels (umbilical arteries and vein) with subsequent fetal hypoxia or by the blood loss within the cord itself with anemia, leading to perinatal asphyxia and stillbirth [1].

In our analysis of 11 cases, 2 cases showed evidence of chorioamnionitis [7, 8], 1 case revealed single umbilical artery and marginal cord insertion with spontaneous avulsion of the umbilical artery [5], 1 case was secondary to composite heterozygous congenital factor VII deficiency, and in 7 cases no pathological condition was reported [3, 4, 9–11].

### 3.2. Diagnosis

The diagnosis is usually made postnatally, but in some cases it can be made by Doppler ultrasound scan prenatally, assessing the cord and the blood flow in the umbilical vessels [11, 12]. Cord hematomas can arise during pregnancy which can lead to fetal death [8], or may occur, more frequent, during labour giving rise to fetal distress and requiring immediate fetal delivery. Of the nine live born cases discussed in this review, 7 presented at term of gestational age, 2 preterm [3–5, 7, 9–11]; moreover, 7 cases presented with a complaint of decreased fetal movement [3–6, 8]. Abnormal fetal heart monitor tracing has been described in 6 cases [3–6]. In 3 cases, there were no changes in fetal movements or abnormalities in fetal heart rate. Stillbirth that occurs in the antenatal period is more difficult to explain than that occurring intrapartum since it is difficult to ascertain a cause of antenatal stillbirth [8].

Detailed physical examination of the placenta and cord confirmed the presence of the hematoma in all 11 cases described. During macroscopic examination, umbilical cord may have abnormal appearance with dark red discoloration and markedly increased thickness [10]. It may have a darkish bulge and a bluish discoloration [9]. Hystopathological examination of the placenta and cord confirmed the presence of hematoma and showed evidence of chorioamnionitis in two cases [7, 8]. The hystological examination of the tract of cord affected by the hematoma may show perivascular hemorrhagic infiltration, umbilical vessels compressed by the hemorrhagic effusion, fissures of the venous wall, alterations of the intima and middle tunica with the vessels wall markedly thinned by the reduction of the muscular component and also moderate inflammatory leukocytic infiltration of the umbilical vascular walls [12].

Autopsy plays an important role in investigating the cause of stillbirth that occurs in the antenatal period.

### 3.3. Case Report

A 29-year-old multipara woman, with an uncomplicated pregnancy, presented at 41 weeks and 3 days of gestation for elective labour induction. The patient showed Grade 1 obesity (BMI of 30 kg/m^2^).

Labour was induced with a controlled-release hydrogel pessary containing 10 mg prostaglandin E2. The patient was placed on continuous fetal heart rate (FHR) monitoring. After 24 hours from the labour induction, the Bishop score was unchanged and the vaginal insert removed. 3 hours later, the induction continued with intravenous injection of oxytocin 10 UI. After approximately 1 hour, spontaneous rupture of membranes with amniotic clear fluid was observed.

8 hours after induction patient delivered a hypotonic, with no evidence of cardiac activity, male newborn. FHR corresponded to type 1 and 0 of Piquard criteria during the second stage of the labour.

Venous pH at birth was 7.11 (base excess, 16.2 mMol/L), and arterious pH was 6.96 (base excess, 14.2 mMol/L). Apgar score at 1 minute was 0. After 40 minutes of continuous resuscitation, the fetus was still asystolic, and it was therefore decided to stop the resuscitation efforts.

The gross examination of the placenta and of the umbilical cord revealed the presence of blackish-reddish material in the proximity of the placental insertion measuring approximately 3 cm. The umbilical cord presented vascular ectasia at 18 cm from the placental insertion. An hematoma of the cord was noted at 34 cm from the placental insertion; the hematoma was described as an infiltrate of 2 cm, in the tones of black and red, extended to the whole umbilical cord section.

The histological examination of the cord highlighted oedema of Wharton's jelly, circumscribed hematic infiltrates, marked venous ectasia with delamination and hematic infiltration of the venous walls, extensive hemorrhage of Wharton's jelly within the whole portion of the cord. The lumen of the vein was completely occluded by coagulated hematic material.

The histological examination of the placenta highlighted intense vascular congestion of villi and hematic infiltrates as for intervillous hematomas.

Measurements of crown heel, crown rump, head circumference, foot length and weight indicated a regular intrauterine development.

The internal gross examination and the hystopathological examination of lung tissue revealed elements indicating physiological respiration in presence of FHR. The above pattern confirmed that the fetus started the respiratory activity after being delivered before dying.

## 4. Discussion

The umbilical cord is called the fetal life line, and it is the vital link between the fetus and placenta. Various abnormalities are observed in the morphology and pathology of the umbilical cord but knowledge of them is quite poor.

A considerable number of stillbirths that are thought to be unexplained may be attributable to placental or cord pathologies. UCH can compromise the maternal-fetal circulation by compressing the vessels or because of the blood loss within the cord itself, leading to perinatal asphyxia and stillbirth [1].

Stillbirth can occur either antenatally or perinatally, but sometimes UCH is uneventful. In our case, the stillbirth was peripartum; the results from external inspection, hystopathological examination, and autopsy suggest the manifestation, before death, of a hyperacute asphyctic mechanism. Furthermore, macro- and microscopic analyses of the umbilical cord revealed pathological alterations indicating an acute trauma with compression, vascular laceration, and hemorrhagic infiltration.

This must be due to the occurrence of mechanical compression of the umbilical cord during labour, with acute interruption of the fetoplacental circulation. The cause of death is therefore attributable to an intrauterine asphyxia caused by acute mechanical compression of the umbilical cord, difficult to detect antenatally.

## 5. Conclusions

Cord accident (compromised umbilical blood flow) as a cause of stillbirth is underreported, mainly due to a lack of diagnostic criteria.

A complete fetopathological examination can state causality between hematoma and stillbirth, exclude another fetal or placental cause of death and consequently reassure the parents for the prognosis of another pregnancy. The issue of hematoma-related complications is also important because of its medicolegal aspect since litigation may occur. Timing of delivery should be based on gestational week as long as the fetus shows well-being signs. Preterm or urgent delivery should be performed in case of fetal distress or reduced movements.

Because of the rarity of this condition, every new case of UCH should be reported in order to improve the knowledge of predisposing factors, prenatal diagnosis, and clinical management.

## Figures and Tables

**Table 1:** Case reports described in literature.

Authors	Age/parity	Gestational age (wk)	Antenatal course	Mode of delivery	Macroscopical lesion	Hystopathological examination	Fetal outcome
Towers et al. [3]	23/1	31	Decreased fetal movements for 18 hours	Cesarean	Umbilical cord hematoma 3 × 2 cm	Hematoma associated with umbilical vein. Thrombotic material was seen within the vein, but the vein was not totally occluded; the umbilical arteries were compressed to the side but patent.	None/AS 7 at 1 min and 9 at 5 min
	22/0	40	Decreased fetal movements for 30 hours	Cesarean	Umbilical cord hematoma 4 × 2 cm	Vein/arterial lumens were compressed but both patent.	None/discharged well at follow up at 18 months/AS 2 at 1 min, 6 at 5 min and 8 at 10 min
	39/1	38	Absent fetal movements for 14 hours	Cesarean	Umbilical cord hematoma 3 × 2 cm	Vein/umbilical arteries appeared patent.	None/AS 8 at 1 min and 9 at 5 min

Barbati et al. [4]	44/1	40	Reduction of fetal movements, FHR with severe reduced variability of <5 bpm and late decelerations	Cesarean	Large cord hematoma (5 × 3.7 × 2.6) at 3 cm from the fetal insertion	Two arteries and one vein with no other abnormalities in the form of knots and loops. Extravasation of blood into the surrounding Wharton's jelly caused by the rupture of a dilated umbilical artery	Tachypnea, cyanosis, and anemia without any other physical or neurological damage/AS 6 at 1 min and 9 at 5 min

Kumar et al. [5]	31/1	36	Two vessel-cord, right pelvic kidney, decreased fetal movement for 12 hours	Cesarean	Marginal umbilical cord insertion, avulsed umbilical artery rupture: single artery is shown to be ruptured at the site of cord insertion to the placenta.	Fetal branch artery ruptured with a vessel wall significant for mild acute inflammation and necrotic muscle cells.	Tachycardia, tachypnea, and polyuric acute kidney failure secondary to cortical-sparing acute tubular necrosis; discharged well at 14 day/AS 3 and 5 at 1 and 5 min

Jouannelle et al. [6]	Not given	At term	Decreased fetal movements, fetal heart decelerations	Cesarean	Massive umbilical cord hematoma at the skin junction, with cord compression	Not given	Baby was flat/AS 0 at 1 min, 3 at 5 min, 7 at 10 min. Postnatal evolution: coma, total hypotonia, no archaic reflex, and hypoxic-ischemic encephalopathy. The newborn died of multiorgan failure on day 6 of life.

Tonni et al. [7]	19/0	At term	Loss of fetal heart lasting 90 seconds, at birth	Vaginal	Fresh hematoma in the cord	A rupture in the wall of the umbilical vein with discontinuity in the layers of the subintimal and internal elastic lamina. One umbilical artery presented peripheral dissection, subintimal myxoid degeneration, and widespread disruption of the elastic fibers; marked reduction in myofibroblasts in Wharton's jelly. Amniotic band at umbilical cord insertion into the chorionic plate, markedly congested chorionic vessels with dispersed distribution and convoluted decourse. The membranes had diffuse chorioamnionitis (*E. coli* infection).	Sever mixed acidosis, fetal anemia, and severe HIE/AS 3 at 1, 5, and 10 min Follow-up at the age of 4 years: spastic tetraplegia, seizures, central deafness, and blindness

Abraham et al. [8]	27/multipara	35	Decreased fetal movements (ultrasound scan confirmed fetal death)	Vaginal	Central cord insertion. Umbilical cord had 4-5 sausage shaped swellings suggestive of cord hematoma of varying sizes all along the length of the cord with the largest measuring 6 × 3 cm	Cord had multiple swellings suggestive of umbilical cord hematoma. Chorioamnionitis	Stillbirth

McAdams and Chabra [9]	32/1	At term	Uneventful	Vaginal	Umbilical cord hematoma	Not given	None

Hooper and Sebire [10]	Not given	At term	Uneventful	Vaginal	Umbilical cord proximal to the baby has dark red discoloration and increased thickness, measuring 4.5 in diameter at the widest part.	Not given	None

Arora et al. [11]	Not given	39	Uneventful	Vaginal	A 4 cm and 2 cm wide reddish purple, nontender swelling in the cord proximal to the level of the skin	Not given	None AS 8 and 9 at 1 and 5 min
