# Corrigendum to “Umbilical Cord Hematoma: A Case Report and Review of the Literature”

**DOI:** 10.1155/2018/1405281

**Published:** 2018-08-01

**Authors:** Gennaro Scutiero, Giulia Bernardi, Piergiorgio Iannone, Luigi Nappi, Danila Morano, Pantaleo Greco

**Affiliations:** ^1^Department of Morphology, Surgery and Experimental Medicine, Section of Obstetrics and Gynecology, Azienda Ospedaliero-Universitaria S. Anna, University of Ferrara, Via Aldo Moro 8, 44121 Cona, Ferrara, Italy; ^2^Department of Medical and Surgical Sciences, Institute of Obstetrics and Gynecology, University of Foggia, Viale L. Pinto, 71100 Foggia, Italy

In the article titled “Umbilical Cord Hematoma: A Case Report and Review of the Literature” [1], the first and last names of the second author were reversed. The revised authors' list is shown above.
